# GDF11 downregulates FOXP3 in T-cell acute lymphoblastic leukemia-derived cells and associates with restraining aggressiveness

**DOI:** 10.32604/or.2025.064899

**Published:** 2025-07-18

**Authors:** MELISSA SáNCHEZ-RODRíGUEZ, ROBERTO LAZZARINI-LECHUGA, VERóNICA SOUZA-ARROYO, LETICIA BUCIO-ORTIZ, ROXANA U. MIRANDA-LABRA, MONSERRAT GERARDO-RAMíREZ, ARACELI PáEZ-ARENAS, MOISES VERGARA-MENDOZA, MARíA CONCEPCIóN GUTIéRREZ-RUIZ, ALEJANDRO ESCOBEDO-CALVARIO, LUIS E. GOMEZ-QUIROZ

**Affiliations:** 1 Área de Medicina Experimental y Traslacional, Departamento de Ciencias de la Salud, DCBS, Universidad Autónoma Metropolitana-Iztapalapa, Mexico City, 09340, Mexico; 2 Posgrado en Biología Experimental. DCBS, Universidad Autónoma Metropolitana-Iztapalapa, Mexico City, 09340, Mexico; 3 Departamento de Biología de la Reproducción, DCBS, Universidad Autónoma Metrolitana-Iztapalapa, Mexico City, 09340, Mexico; 4 Laboratorio de Medicina Experimental, Unidad de Medicina Traslacional IIB/UNAM, Instituto Nacional de Cardiología Ignacio Chávez, Mexico City, 14080, Mexico; 5 Laboratorio de Cardiología Traslacional, Unidad de Medicina Traslacional, IIB/UNAM, Instituto Nacional de Cardiología Ignacio Chávez, Mexico City, 14080, Mexico; 6 Departamento de Enfermedades Infecciosas, Instituto Nacional de Ciencias Médicas y Nutrición Salvador Zubirán, Mexico City, 14080, Mexico

**Keywords:** Growth differentiation factor 11 (GDF11), Leukemia, Cancer, Jurkat cells, Forkhead-box-protein P3 (FOXP3)

## Abstract

**Background:**

Growth differentiation factor 11 (GDF11), a transforming growth factor-beta superfamily member, is a crucial protein involved in many differentiation processes in embryogenesis and morphogenesis, and it has been extensively characterized due to its capacity to target poorly differentiated cells, including transformed or cancer cells.

**Aim:**

In the present work, we aimed to describe the effects on migration, proliferation, and metabolism in the T-cell acute lymphoblastic leukemia-derived cell line Jurkat.

**Methods:**

Based on previous evidence, we analyzed metabolic changes exerted by GDF11 and its relationship with the aggressive phenotype.

**Results:**

We found a profound impact on mitochondrial metabolism and reactive oxygen species content; these were related to a decrement in the expression of the transcription factor forkhead-box-protein P3 (FOXP3), which is highly involved in aggressiveness in leukemia cells; this was verified by a decrement in invasion capacity exhibited by the Jurkat cells under the GDF11 treatment.

**Conclusion:**

The results position the GDF11 response as a good alternative in the search for new therapeutic options for these diseases.

## Introduction

The growth differentiation factor 11 (GDF11) is a family member of the transforming growth factor-beta (TGF-b). It is reported to exert key activities in morphogenesis [1] but is also associated with muscle regeneration, targeting satellite cells [2], and protective properties in heat damage [3]. Although it has been involved in many controversies, we believe that those disagreements are, in part, due to the degree of differentiation of the target cell [4]. This is particularly relevant in cancer cells. Many reports have pointed out the effects of GDF11 on cancer [5]. Our group has characterized the antitumoral properties in liver cancer, decreasing proliferation, migration, clonogenicity capacity, spheroid formation [6], and a decrease in aberrant metabolism [7]; others have reported similar findings in other types of cancer, including breast [8], pancreas [9], and esophageal [10], among other solid tumors.

Considering that GDF11 impacts poorly differentiated cells, we decided to explore the effects of this growth factor on cells derived from human leukemia. There is poor evidence regarding the GDF11 in the biology of leukemia cells. Recently, Aslan and collaborators published that patients with acute myeloid leukemia presented increased serum levels of GDF11 [11], but this study was descriptive, providing only correlative evidence.

Hematological malignancy T-cell acute lymphoblastic leukemia (T-ALL) is a subtype of acute lymphoblastic leukemia (ALL) characterized by an overproduction of poorly differentiated T lymphocytes. It is the most common type of cancer in children [12]. T-ALL accounts for 10% to 15% of pediatric ALL cases and up to 25% of adults [13]. This leukemia is even more aggressive than B-cell ALL and is commonly associated with leukemic and lymphomatous manifestations [14]. There is much evidence for the biology studies of T-ALL and the use of the Jurkat cell line, mainly for apoptosis, T-cell activation, and signal transduction [15]; also, Jurkat cells express the transcription factor forkhead-box-protein P3 (FOXP3), which has been reported to control many pro-oncogenic properties conferring aggressiveness in cancer cells, and poor prognosis in patients [16]. It is implicated in T-cell regulation, activation, and differentiation and is considered a master regulator for developing and functioning CD4+/CD25+ regulatory T-cells [17].

In this research, we focused on characterizing the effects of GDF11 on the expression of FOXP3 and its implication in cancer cell aggressiveness.

## Materials and Methods

Human recombinant Growth Differentiation Factor 11 (GDF11) was from Peprotech (Cat. 120-11, Rocky Hill, NJ, USA); as we previously reported, all treatments with the growth factor were at 50 ng/mL at different incubation times [6,7].

### Cell culture

The Jurkat cell line was purchased from the American Type Culture Collection (ATCC, TIB-152, Manassas, VA, USA). Jurkat cell is an immortalized T lymphocyte cell line originally obtained from the peripheral blood of a patient with T-cell leukemia. Cells were cultured in RPMI 1640 medium (Gibco BRL, Cat. 31800022, New York, NY, USA). Culture used 10% fetal bovine serum (FBS, HyClone, Cat. SH3091003, Logan, UT, USA), 100 U/mL ampicillin, and 100 µg/mL streptomycin (Thermo Fisher Scientific, Cat. 15070063, Waltham, MA, USA). Cells were maintained at 37°C in a 5% CO_2_ and 90% humidity atmosphere. Cells were plated in plastic culture dishes (Corning, Cat. 353037, New York, NY, USA). The cell line was mycoplasma-free.

### Protein isolation and quantification

Protein extraction was performed using M-PER (Thermo Fisher Scientific, Cat. 78505, Boston, MA, USA) lysis buffer containing protease inhibitors (Complete, Sigma-Aldrich, Cat. 11836170001) and phosphatase inhibitors (PhosSTOP, Sigma-Aldrich, Cat. 4906845001). Protein quantification was performed using the bicinchoninic acid (BCA) kit (Pierce, Thermo Fisher Scientific, BCA1-1KT, Waltham, MA, USA) according to the manufacturer’s instructions. We used 60 mg of protein in the Western blot assays.

### Western blot

Western blotting was performed following the protocol previously reported [6]. Briefly, proteins were loaded onto precast 4–20% gels (Invitrogen, Cat. WTG42012BOX, Waltham, MA, USA), transferred to polyvinylidene difluoride membranes (PVDF, Thermo Fisher Scientific, Cat. 88518), and probed with primary antibodies as listed in Table A1. The membranes were incubated with the corresponding horseradish peroxidase-conjugated secondary antibody. Immunoreactive bands were identified with ECL-Plus Western blotting substrate (Thermo Fisher Scientific Pierce, Cat. 32134). Images were analyzed using ImageJ v. 1.54p software (National Institutes of Health, Bethesda, MD, USA).

### Cell viability

Cell viability was assayed by flow cytometry using the BD FACSCalibur flow cytometer (Becton Dickinson, Life Sciences, Franklin Lakes, NJ, USA); we used the LIVE/DEAD fixable green stain (Thermo Fisher Scientific, Cat. L23101) following the manufacturer’s instructions. Data were analyzed using FlowJoX (Version 10.X, Becton Dickinson Life Sciences).

### Real-time cellular metabolism determination

The real-time oxygen consumption rate (OCR) was assayed with the Seahorse XF^e^24 Flux Analyzer (Agilent Technologies, Santa Clara, CA, USA), as we recently reported [18], and according to the manufacturer’s instructions. Briefly, 1 × 10^6^ Jurkat cells were pre-treated using the recombinant human GDF11 every 24 h until 72 h in FBS media. After incubation, the cells were harvested immediately and replated at 175,000 cells/well in an XFe24-well plate format with a previous Cell-Tak (Corning) matrix treatment per well. We conducted the XF Cell Mito Stress Test (Agilent Technologies, Cat. 1030 15-100); cells were incubated with unbuffered assay media (XF Base RPMI Media with 10 mM glucose, 2 mM L-glutamine, and 1 mM pyruvate) followed by sequential injection of 0.5 μM oligomycin, 1 μM carbonyl cyanide-4 (trifluoromethoxy) phenylhydrazone (FCCP), and 0.5 μM antimycin A plus Rotenone. According to the previously referred protocol, OCR measurements were normalized with total cell protein.

### ROS determination

Jurkat cells were centrifuged and incubated (37°C) in the dark with dihydroethidium (DHE, 5 μM, Invitrogen, D11347) using PBS for 15 min. Superoxide radicals were detected using a BD FACSCalibur (Becton Dickinson Life Science) flow cytometer. Excitation and emission wavelengths were 518 and 606 nm, respectively [19]. We acquired 10,000 events in duplicate. Data were analyzed using FlowJo™ Software (Becton Dickinson Life Sciences).

### FOXP3 determination and nuclear colocalization

Cells were treated with GDF11 for 24, 48, and 72 h; after that, they were fixed using PFA (4%) and permeabilized using Cyto-Fast Fix/Perm buffer Set (BioLegend, Cat. 10482735, San Diego, CA, USA) on ice for 15 min. The anti-FOXP3 was applied according to the Table A1. The antibody was conjugated with phycoerythrin (PE). TGF-b (20 ng/mL for 24 h. Peprotech, Cat. 100-21,Cranbury, NJ, USA) was used as a positive control of FOXP3 expression. Flow cytometry analysis was performed using 10,000 events by duplicate in a BD FACSCalibur flow cytometer (Becton Dickinson Life Science), and data obtained were analyzed using FlowJoX (Becton Dickinson Life Science).

For confocal colocalization of FOXP3 in the nucleus, cells were treated at different times with GDF11; after that, cells were incubated with the mAb anti-FOXP3 (Thermo Fisher Scientific, Cat. 236A/E7) overnight, next day, cells were concentrated by centrifugation and incubated 5 min with 1 μg/mL 4′,6-diamidino-2-phenylindole (DAPI) (Sigma-Aldrich, Cat. D9542). Images and colocalization were determined using a multi-photon confocal microscope (Carl Zeiss LSM-780 NLO, Oberkochen, Germany) and the Zen Analyzer software (Carl Zeiss V3.11).

### Cell proliferation

Cell proliferation was addressed by the colorimetric cell proliferation and cytotoxicity assay, Cell Counting Kit-8 (CCK-8; Sigma-Aldrich, Cat. 96992), following the manufacturer’s instructions. Treatments with 20 mM cisplatin, for 24 and 48 h, were used as a positive control for cell damage.

### Invasion assay

Jurkat cell invasion was assessed using Boyden’s chamber (Thermo Fisher Scientific, pore diameter 8 µm, Cat. 140629). We added 1 × 10^6^ cells, treated or not with GDF11, in 600 µL of RPMI medium previously covered with 200 µL of Matrigel Matrix (Corning Matrigel, hESC-Qualified Matrix, Cat. CLS354277). The lower chamber had the same medium supplemented with 20% FBS. Chambers were incubated overnight, and we counted cells in the lower chamber. We used colchicine (Col, 10 mM, Sigma-Aldrich, Cat. C9754) as a negative control, and a chamber was Matrigel-untreated to observe the migratory process through the filter as a technical control (C).

### Statistical analysis

Data are presented as mean ± SEM for at least three independent experiments; each experiment was conducted in triplicate. An analysis of variance (ANOVA) was used to compare the means of different groups, followed by multiple comparisons by the Tukey test. Graph Pad Prism software (Dotmatics, CA, USA) version 8 for macOS was used. Differences were considered statistically significant when *p* < 0.05.

## Results

### GDF11 activates the canonical signaling pathway in Jurkat cells

**Figure 1:**
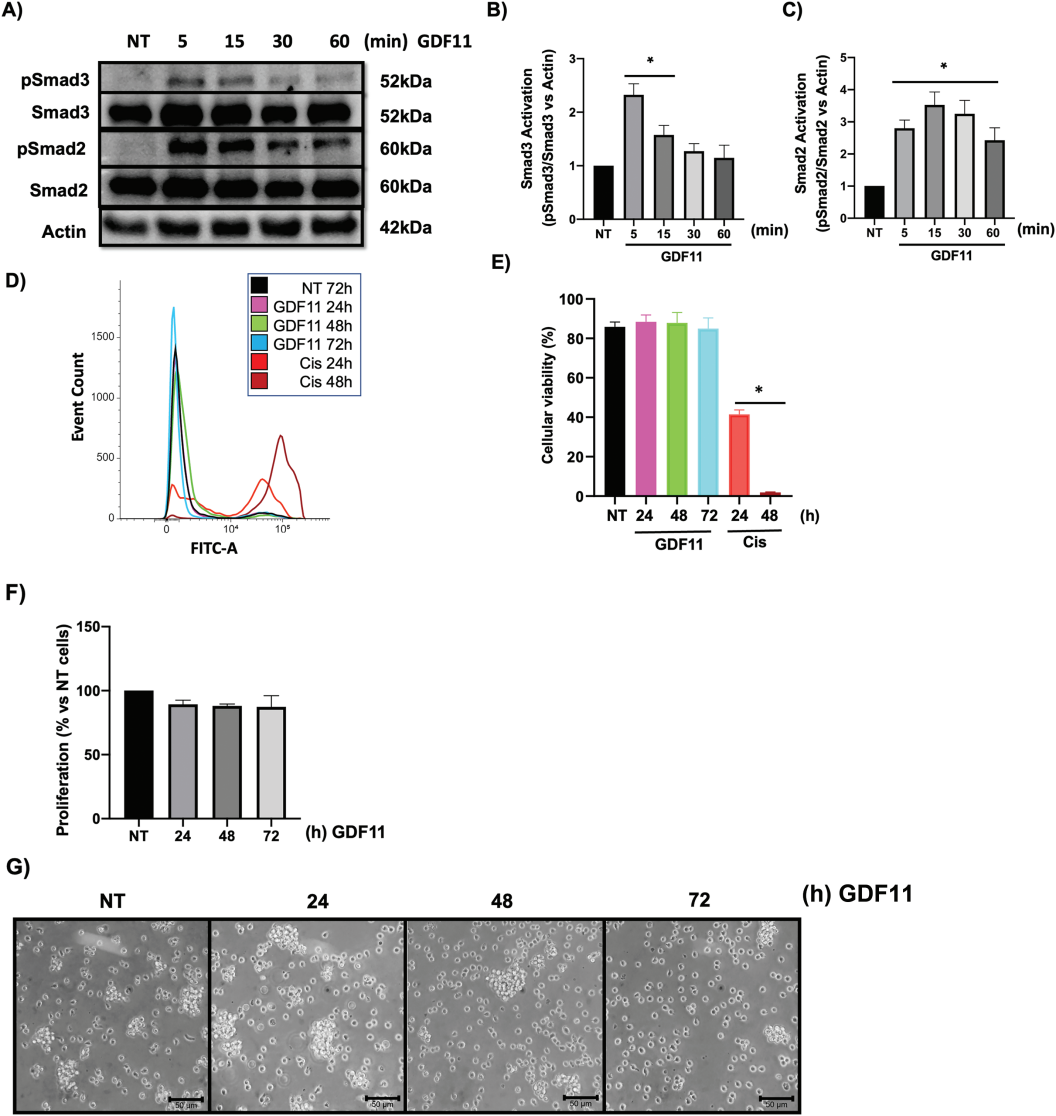
Jurkat cells respond to GDF11 treatment, activating Smad2 and Smad3 without affecting cell viability. Cells were treated with GDF11 (50 ng/mL) at various time points. (A) Western blot analysis of the Smad2 and Smad3 phosphorylation. Actin was used as a loading control. Densitometric analysis of (B) Smad3 and (C) Smad2. Each column represents the mean ± SEM of at least three independent experiments in triplicate. Cell viability was assessed by flow cytometry according to the protocol outlined in Materials and Methods. (D) Overlay histogram of the stained population; (E) Percentage of viable cells. Cisplatin (20 mM) at 24 and 48 h was used as a positive control; (F) Cell proliferation determined by CCK-8; Each column represents the mean ± SEM of at least three independent experiments in triplicate; (G) Cell morphology observed by light microscopy. Representative images of at least three independent experiments. *p < 0.05 vs. NT.

First, to corroborate the specific response of Jurkat cells to GDF11, we treated cells with the growth factor at early times (5–60 min). We evaluated the activation of Smad proteins, the canonical signaling pathway in the TGF-β family. Phosphorylation of Smad2 and Smad3 was assessed by Western blot (Fig. 1A). The results show that both Smad3 (Fig. 1B) and Smad2 (Fig. 1C) were activated by GDF11, peaking at 5 min and progressively decreasing over time. We could not detect any effect on cell viability, as judged by the flow cytometry assay, at late incubation times (Fig. 1D,E).

As previously reported, GDF11 has biological effects on cancer cells during prolonged incubation, particularly at 72 h; we treated cells for 24, 48, and 72 h. We did not observe changes in cell proliferation or morphology (Fig. 1F,G), which partially agrees with our previous results [6].

### Metabolic effects induced by GDF11

**Figure 2:**
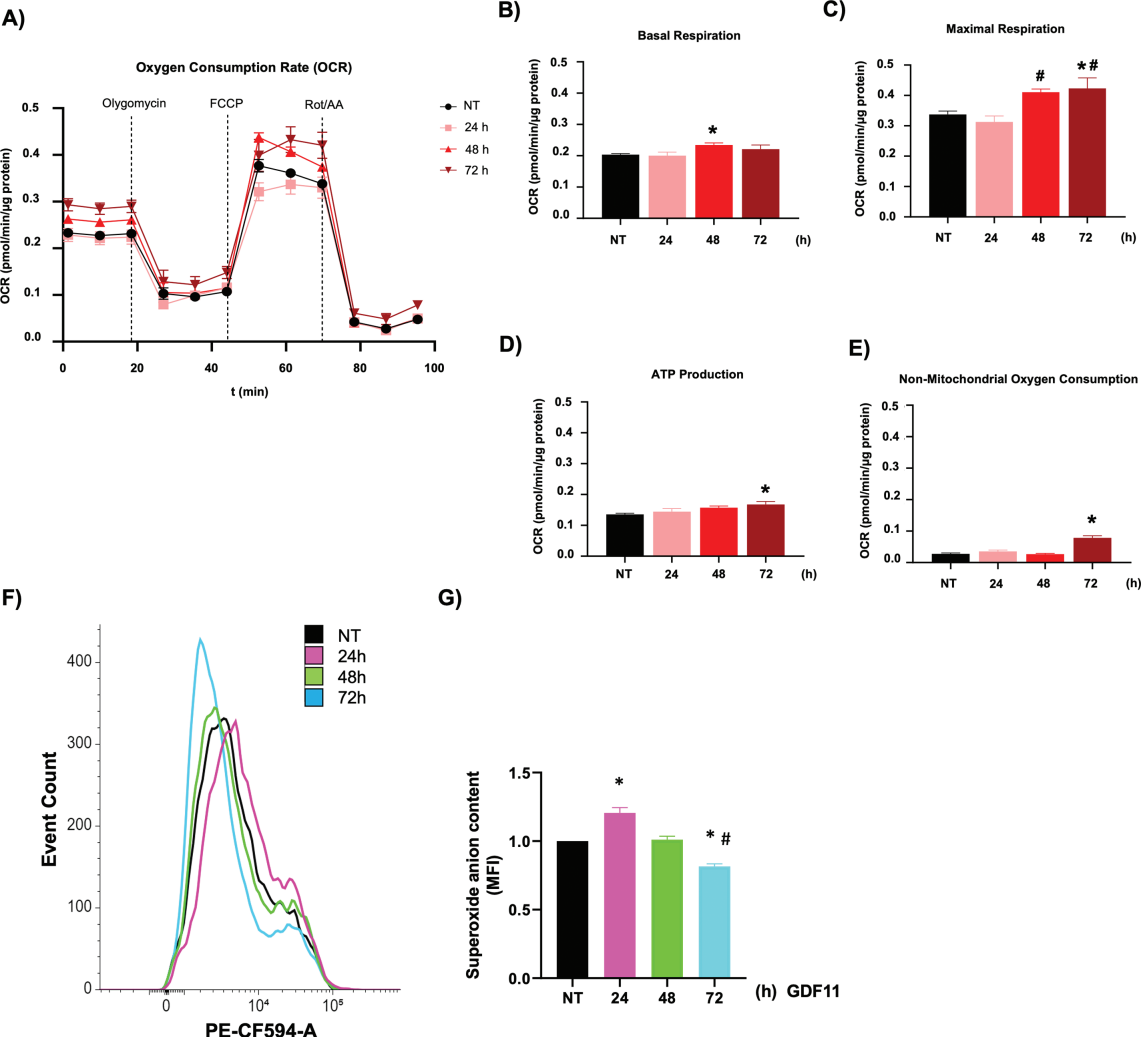
GDF11 promotes bioenergetic rewiring in Jurkat. (A) Mitochondrial metabolic activity was determined by oxygen consumption rate (OCR) using a Seahorse XF24e flux analyzer. The processed data were depicted as (B) basal respiration, (C) maximal respiration, (D) ATP production, and (E) non-mitochondrial oxygen consumption. (F) Overlay histogram of the DHE fluorescence; (G) The mean fluorescence intensity (MFI) of DHE. Each column represents the mean *±* SEM of at least four independent experiments. *p < 0.05 vs. NT; ^#^*p* < 0.05 vs. 24 h.

Considering our previous findings in hepatocellular carcinoma cells, where we found changes in metabolic parameters, we performed a real-time metabolism study using the Seahorse technology [7]. We focused on mitochondria metabolism, exploring the oxygen consumption rate (OCR, Fig. 2A); we observed a clear separation in the bioenergetic behavior of the cells during the time of the study; profound data analysis revealed that treatment with GDF11 leads to metabolic modulation in the mitochondria, making more efficient the metabolism of these organelles, as indicated by basal respiration after 48 h (Fig. 2B), and maximal respiration at 48 and 72 h (Fig. 2C), reflected in the production of ATP at 72 h (Fig. 2D); and linked to an increment in non-mitochondrial oxygen consumption (Fig. 2E). These data demonstrate that GDF11 is involved in bioenergetics changes more than glycolytic metabolism, where we could not find any difference in Seahorse analysis (Data not shown).

### GDF11 modulates reactive oxygen species

The metabolism analysis suggests changes in the cell’s redox state. We analyzed the ROS content, observing a biphasic response of superoxide anion formation, increasing at 24 h of treatment and decreasing at 72 h, regarding untreated cells (Figs. 2F,G). The data indicate a possible signal modulation for different pathways in T cells, including mitochondria, led by ROS.

### GDF11 decreases FOXP3 expression and reduces nuclear localization

**Figure 3:**
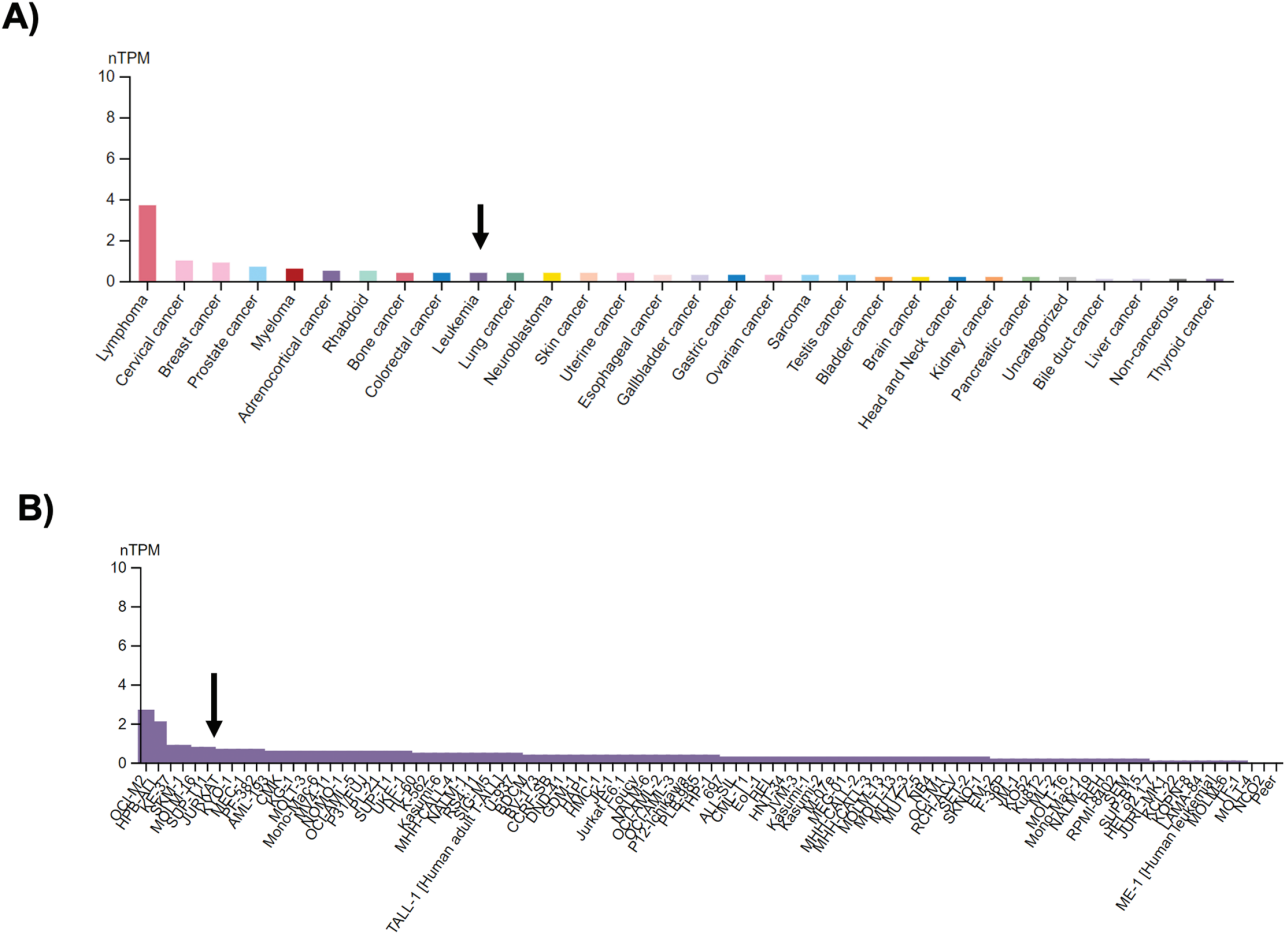
FOXP3 expression in cancer and human cancer-derived cell lines. (A) FOXP3 expression by cell category, according to the human protein atlas (proteinatlas.org). The arrow shows the expression of FOXP3 in Leukemia; and (B) FOXP3 expression in 93 leukemia cells, the arrow shows the Jurkat cell line. nTPM, normalized transcript per million.

It has been reported that ROS exerts a regulatory response in key transcription factors related to transformed T lymphocytes, such as FOXP3 [20,21]; the human protein atlas (proteinatlas.org) shows the high relevance of FOXP3 expression in different cancer cells, including leukemia (arrow, Fig. 3A), and Jurkat cells positions as a cell line with high expression of this transcription factor (arrow, Fig. 3B) [22].

**Figure 4:**
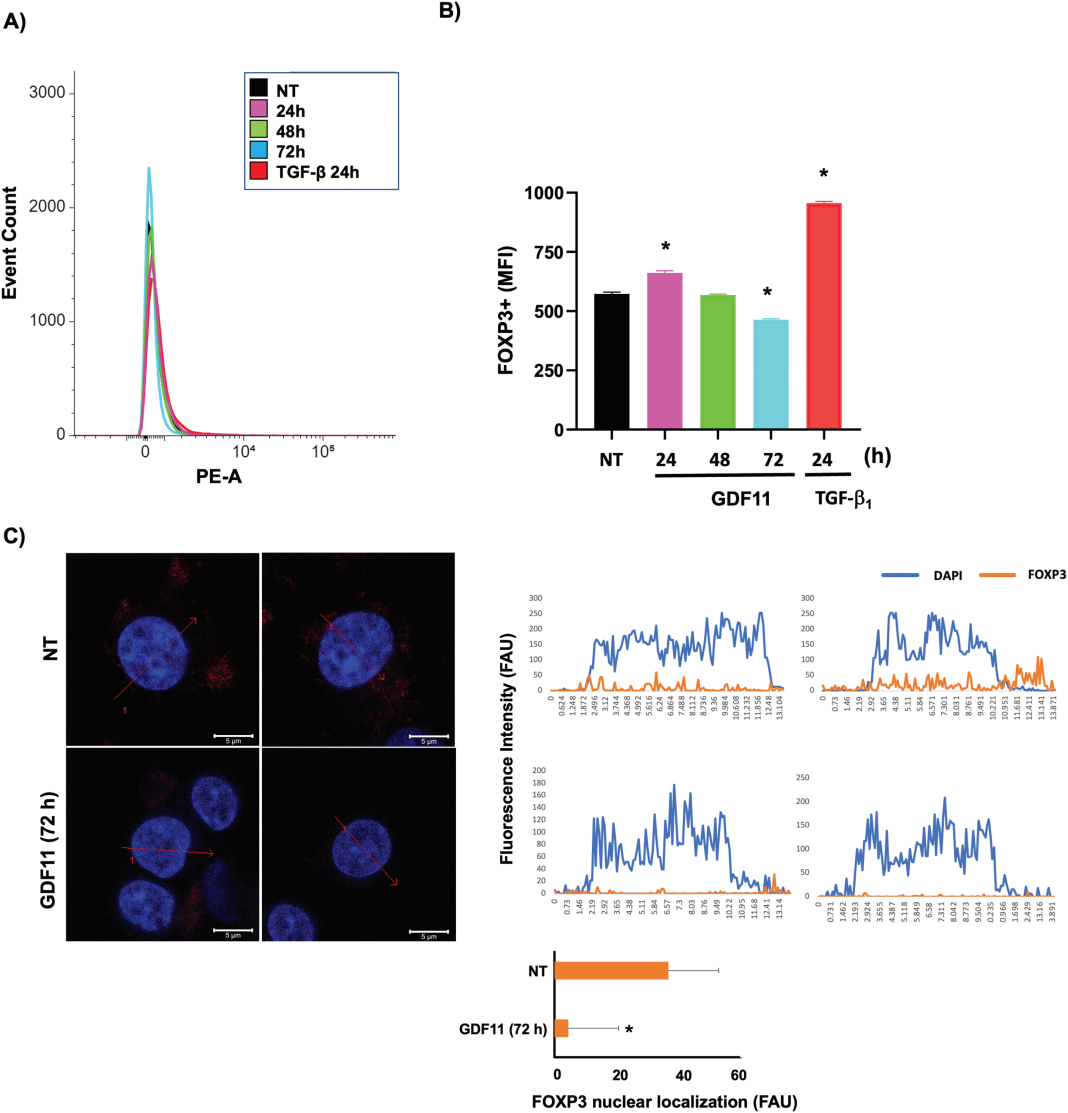
FOXP3 expression and nuclear localization in Jurkat cells. (A) Overlay histogram of the stained population; (B) The mean fluorescent intensity (MFI) of FOXP3-positive cells. (C) Nuclear co-localization of FOXP3 determined by confocal microscopy; intensity profiles of four different experiments of each cell group obtained with the Zen software, along a straight line (red arrow) crossing the nucleus of representative cells of FOXP3 (red) and DNA staining by DAPI (blue). The figure shows two representative images of each experiment. Histogram depicting the average quantification of fluorescence intensity plotted as fluorescence arbitrary units (FAU). *p < 0.05 vs. NT.

Based on this evidence, we decided to explore the expression of this relevant protein in cells under GDF11 treatment. Fig. 4A,B shows that the FOXP3 content, determined by flow cytometry, follows a biphasic response, increasing at 24 h and decreasing to control values at 72 h; the TGF-b (20 ng/mL for 24 h) was used as a positive control of FOXP3 expression. This data strongly suggests a ROS-dependent regulation. To gain more confidence, we performed a colocalization study of FOXP3 in the cellular nucleus; Fig. 4C shows the basal colocalization of the transcription factor FOXP3 (anti-FOXP3, red) with the DNA (DAPI, blue), the treatment for 72 h with the GDF11 induces a decrease of the presence of FOXP3 in the nucleus. These data suggest a decrement in the content and nuclear localization of FOXP3 induced by the GDF11. FOXP3 is a critical transcription factor for leukemia cells; this protein is reportedly closely associated with high aggressiveness [23].

### GDF11 limits migration capacity in Jurkat cells

**Figure 5:**
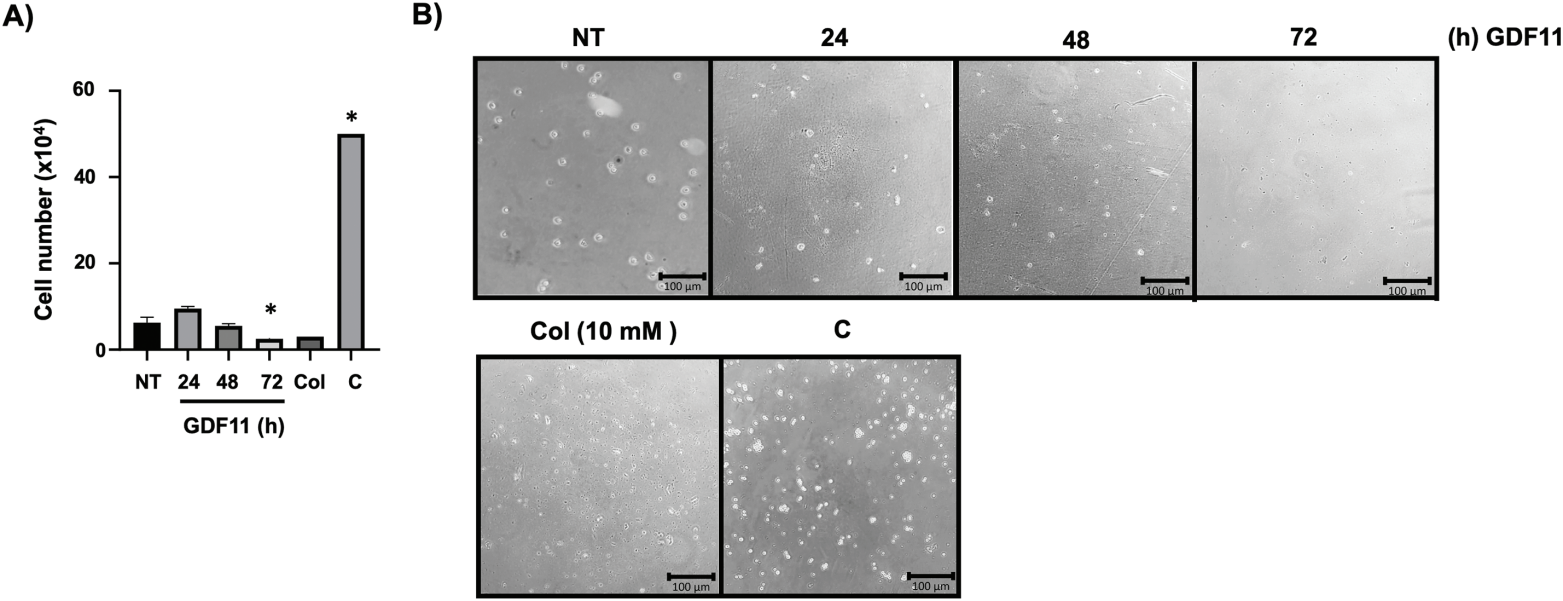
GDF11 limits migration and invasion capacity in Jurkat cells. (A) Migration and invasion assay determined by Boyden’s chambers. Cells were counted in the lower chamber at 24 h of the study. (B) Representative images of the cells in the lower chamber. Col (colchicine, 10 mM) as negative control, and (C) as technical control without Matrigel. Each column represents the mean *±* SEM of at least three independent experiments in triplicate. *p < 0.05 vs. NT.

To support the idea that GDF11 could induce a cellular response tending to decrease aggressivity in the leukemia cells, we assessed the migratory and invasive capacity of the cells under GDF11 treatment using Boyden’s chambers. Fig. 5A,B shows a time-dependent decrement in the migratory capacity, indicating a decrement in cell aggressiveness.

## Discussion

GDF11 has been implicated in many controversies, in part because foundational investigation in the biology of this growth factor was based on non-optimized research tools, for example, antibodies that could detect the GDF8 or myostatin, a close family member of the GDF11, and also because the degree of differentiation or stemness could be determinant in the biological response of the growth factor [4]. Considering these issues, exploring the effects of GDF11 in cancer biology could yield more insights.

It has been proposed that GDF11 can exert antitumoral effects in many cancer cell lines, including those from the liver [6,7], the breast [8], the esophagus [10], and the pancreas [9], among others; in fact, there is a clear association in survival in patients with high expression of GDF11 compared to those with low expression [5].

Previous works show that GDF11 exerts antitumoral effects in cancer cells, mainly targeting the aberrant metabolism of lipids and bioenergetics [7,24]. This was associated with the decrement of critical markers of aggressivity, such as spheroid formation, proliferation, clonogenicity capacity, migration, and mesenchymal to epithelial transition [6].

In the present study, we aimed to determine the relevance of GDF11 in T-cell acute lymphoblastic leukemia-derived cells using Jurkat cells. Due to the extensive use of this cell line, Jurkat cells serve as an effective model for exploring biological relevance, particularly regarding the significance of a critical disease like leukemia.

Our results demonstrated that Jurkat cells responded by activating the canonical signal transducers of Smad proteins, which agrees with many studies indicating the activation of Smad3 and Smad2 [6,24,25]. Proliferation and survival were not affected, but we found a profound change in mitochondrial metabolism, consistent with an anti-lipogenic response and a decrement in aggressiveness in cancer cells. It is reported that cancer cells can sequester mitochondria by overloading the organelle with cholesterol; this confers apoptosis resistance and aggressiveness but is detrimental to mitochondrial ATP production [26,27], to a more glycolytic metabolism.

Our data agree with these observations; we report an improvement in mitochondrial function judged by the Seahorse analysis of the OCR. Cruz and collaborators [28] show that oxidative phosphorylation (OXPHOS) is elevated in T-ALL cell lines, such as CCRF-CEM and Jurkat cells, compared to normal B and T cells. The basal OCR and ATP linked to respiration indicate mitochondrial dependence in T-ALL cells. Higher OCR from mitochondria represents more aggressiveness in leukemia cells due to a specific mechanism. The mitochondria constitutively transfer calcium from the endoplasmic reticulum through the inositol 1,4,5-triphosphate receptor (InsP3R), making all T-ALL bioenergetic cells susceptible to its inhibition. Cruz’s group used Xestospongin B (XeB), the InsP3R inhibitor; this reduced the bioenergetic parameters on T-ALL cells, inducing apoptosis, even though they are more effective on cell death when used in combination with dexamethasone [28]. We hypothesize that Jurkat cells increased OCR as a protective mechanism against cell death. However, this mechanism could indicate a double-edged sword; it may be more susceptible to combination therapies. Other reports using K562 and KCL-22, cells derived from chronic myelogenous and chronic myeloid leukemias, respectively, reported similar findings according to an increment in bioenergetics parameters such as OCR when using Imatinib, a BCR-ABL inhibitor. They reported reduced cell viability and apoptosis due to decreased caspase 3 activity; an increase in bioenergetic metabolism could have advantages and disadvantages [29].

Mitochondrial fitness could activate intrinsic apoptosis by releasing cytochrome c. Our results may suggest that GDF11 could have positive effects when combined with other therapies. On the other hand, mitochondria modulation could drive possible immunomodulatory or immunometabolic responses. Activation led by glycolysis could lead to Th1 (intracellular bacteria), Th2 (parasites), or Th17 (extracellular bacteria) activation in normal lymphocytes, contrary to OXPHOS, which carries out T regulatory (Treg) or memory cell phenotypes [30]. We can not specify a phenotype in the present study because Jurkat cells are not differentiated T cells. Still, we can hypothesize that they are associated with one specific secretome and specific markers such as FOXP3.

Interestingly, we observed a biphasic regulation of ROS content that correlated with the expression of FOXP3, a transcription factor influenced by ROS [21] and related to more aggressiveness [23]. Interestingly, according to The Cancer Protein Atlas, leukemia cells prefer an increment in FOXP3 expression compared to other types of cancer. The Jurkat cells represent one of the most aggressive ALL cell lines. There is evidence that the reduction of FOXP3 increases the survival rate in leukemia patients, and targeting this transcription factor could be a promising therapeutic strategy, even in solid tumors [17]. Our results show that GDF11 successfully decreased the expression of FOXP3, which was associated with a minor presence of the transcription factor in the nucleus. FOXP3 represents a malignant marker in non-cancerous and differentiated lymphocytes, such as Treg cells, that control and induce immunosuppressive properties in the tumor microenvironment [17,31].

A functional study of invasion addressed the impact of the aggressive phenotype; we demonstrated that GDF11-treated Jurkat cells significantly decreased the migratory and invasion capacity. It has been reported that Jurkat cells promote MMP-2 and MMP-9 secretion under pro-inflammatory stimuli [32,33].

Although this study provides evidence that GDF11 decreases some markers of aggressiveness in human-derived leukemia cells, it is important to note that the main limitation is the cellular model. This work presents an alternative approach to implementing pre-clinical studies that advance and consider the lessons learned from GDF11 as a negative regulator of FOXP3 in this type of cancer.

## Conclusion

The present study provides evidence that GDF11 induces a decrease in the aggressiveness of Jurkat cells through a mechanism dependent on mitochondrial metabolic restoration and redox state modulation. This mechanism affects the expression and nuclear localization of the FOXP3 transcription factor. This research suggests that GDF11 could be an exciting target for further exploration in leukemia therapeutic approaches.

## Data Availability

The data supporting this study’s findings are available from the corresponding authors upon reasonable request by any qualified researcher.

## Abbreviations

ALL: acute lymphoblastic leukemia
CD: cluster of differentiation
DAPI: 4′,6-diamidino-2-phenylindole
DHE: dihydroethidium
FCCP: carbonyl cyanide-4 (trifluoromethoxy) phenylhydrazone
FBS: fetal bovine serum
FOXP3: forkhead-box-protein P3
GDF11: growth differentiation factor 11
OCR: oxygen consumption rate
OXPHOS: oxidative phosphorylation
PVDF: polyvinylidene difluoride membranes
ROS: reactive oxygen species
SEM: standard error of the mean
T-ALL: T-cell acute lymphoblastic leukemia
TGF-b: transforming growth factor-beta

## Appendix A

**Table A1:** Antibodies used in the study

Protein	Brand	Catalog number	Dilution
pSmad2	Cell Signaling Technology, Danvers, MA, USA	3108S	1:1000
Smad2	Cell Signaling Technology	5339 S	1:1000
pSmad3	Cell Signaling Technology	9520S	1:1000
Smad3	Abcam Limited, Cambirdge, UK	Ab40854	1:1000
Actin	Sigma-Aldrich, Saint Louis, MO, USA	A2066	1:2000
Anti Rabbit	Abcam Limited	6721	1:10,000
FOXP3	Monoclonal antibody (236A/E7), PE, eBioscience (San Diego, CA, USA)	12-4777-42	1:200
